# Novel Use of PIT Tags in Sea Cucumbers: Promising Results with the Commercial Species *Cucumaria frondosa*

**DOI:** 10.1371/journal.pone.0127884

**Published:** 2015-05-26

**Authors:** Bruno L. Gianasi, Katie Verkaik, Jean-François Hamel, Annie Mercier

**Affiliations:** 1 Department of Ocean Sciences (OSC), Memorial University, St John's, Newfoundland and Labrador, Canada; 2 Society for the Exploration & Valuing of the Environment (SEVE), St Philips, Newfoundland and Labrador, Canada; University of Pennsylvania, UNITED STATES

## Abstract

The lack of a reliable and innocuous mark-recapture method has limited studies that would provide essential information for the management of commercial sea cucumbers. Tagging sea cucumbers is notoriously difficult because of their plastic nature and autolysis capacities. The markers that have so far been tested, mainly on or through the body wall, were either lost rapidly or had major drawbacks (e.g. suitable only for batch identification, requiring complex analysis, causing infections, necrosis, behavioural changes and mortality). The present study explored the efficacy of passive integrated transponder (PIT) tags for individually marking sea cucumbers by assessing retention rates and long-term side effects of tags inserted in previously unstudied tissues/organs. Individuals of the species *Cucumaria frondosa* were tagged in the body wall, aquapharyngeal bulb and at the base of the oral tentacles. They were monitored closely for evidence of stress, infection, change in feeding and spawning behaviour and tag retention rate. Implanting the tag in an oral tentacle to reach the hydrovascular system of the aquapharyngeal bulb achieved the best retention rates in full-size individuals: from a maximum of 92% after 30 days to 68% at the end of the experimental period (300 days). Efficacy was lower in smaller individuals (84% after 30 d and 42% after 300 d). Following a slight increase in cloacal movements for 15 h post tagging, no side effect was noted in sea cucumbers tagged in the aquapharyngeal bulb via the tentacles. Feeding and spawning behaviours were not affected and no signs of infections or abnormal cell development in the vicinity of the tags were observed. This study indicates that marking sea cucumbers with 8.2 mm long PIT tags implanted via the oral tentacle is an effective technique, yielding relatively high retention rates over long periods without any detectable physiological or behavioural effects.

## Introduction

The high demand and high market prices for beche-de-mer (dry body wall) spurred by cultural and social traditions in China have led to the growth of sea cucumber fisheries and, consequently, to the depletion of wild stocks of high-value species all over the world [1–6].

*Cucumaria frondosa* is the focus of an emerging fishery in the Northwest Atlantic, and it has already become one of the predominant commercial sea cucumber species in terms of landed weight [7]. Although growth rates in *C*. *frondosa* are very low in the wild and in captivity [8,9], the species is considered to have potential for aquaculture in the North Atlantic due to its high marketability for food and pharmaceutical products and because much of its life cycle is well documented [7–9]. It is currently being explored as an extractive species for integrated multi-trophic aquaculture [10]. Several studies have been conducted on sea cucumber ecology in the context of conservation and management efforts [1,8,9,11–15]. However, the lack of an easy and reliable technique to mark individuals has hindered tracking and capture-recapture studies, which provide key biological information (e.g. movement and migration patterns, growth, estimates of natural mortality). The development of an effective tagging technique that minimizes tissue damage, stress and infections, while maximising retention rates will be of great value in years to come, as this fishery expands and aquaculture develops. Such a tool will benefit the sea cucumber industry worldwide by allowing fishery-oriented and ecological studies using mark-recapture to examine temporal changes in growth, survival and mortality rates, as well as daily and seasonal migrations, localization of breeding populations and determination of habitat preferences in the field [16,17].

The difficulties in tagging sea cucumbers are attributed to the plasticity of the body wall, lack of hard tissue, high likelihood of expelling foreign materials and the common occurrence of infections and necrosis around the tagged area [17,18]. Most of the techniques tested so far (summarized in Table 1) have yielded limited success and considerable drawbacks [19,20]. External tags such as T-bars or anchor tags, which are inserted through the body wall using a tagging gun, have shown relatively high retention rates in the first month [18]. However, side effects included damage to internal organs, localized necrosis, influx of seawater into the coelomic cavity through the injection hole, evisceration, mortality and high shedding rates in the long-term [6]. Also, the fact that anchor tags hang outside the animal’s body and are usually colourful works to increase tag loss and mortality by predation [17,21]. Scratched and branded numbers, as well as tags glued or sewed on the dorsal epidermis have also been used to mark sea cucumbers, but the incidence of infection following such procedures is very common [17]. In addition, when the lesions caused by scratches and burns do not evolve into necrosis, the marks disappear within weeks as superficial wounds heal [17,22].

**Table 1:** Summary of markers tested in sea cucumbers with retention rates and drawbacks of each tagging technique.

Type	Technique	Species tested	Maximum retention rate	Drawbacks	References
External tags	T-bars (through body wall)	*Actinopyga echinites*	5% after 1 year	Evisceration, mortality	[18]
		*Actinopyga mauritiana*	5% after 1 year	Evisceration, mortality	[18]
		*Actinopyga miliaris*	60% after 8 days	Necrosis, infection	[29]
		*Cucumaria frondosa*	65% after 140 days	Damaged internal organs	[32]
		*Holothuria nobilis*	5% after 1 year	Evisceration, mortality, reduced growth, increased mobility in the field	[18]
		*Holothuria scabra*	5% after 1 year	Evisceration, mortality, reduced growth, increased mobility in the field	[18]
		*Holothuria scabra*	0% after 30 days	Evisceration	[24]
		*Holothuria lessoni* (as *H*. *scabra versicolor*)	5% after 1 year	Evisceration, mortality, reduced growth, increased mobility in the field	[18]
		*Holothuria whitmaei*	50% after 8 days	Necrosis, infection	[29]
		*Parastichopus californicus*	40% after 224 days	Skin sloughing, open sores, mortality, increased mobility in the field	[16]
		*Stichopus herrmanni* (as *S*. *variegatus*)	5% after 1 year	Evisceration, mortality, reduced growth, increased mobility in the field	[18]
		*Thelenota ananas*	5% after 1 year	Evisceration, mortality, reduced growth, increased mobility in the field	[18]
	Scratches/brands (on body wall)	*Actinopyga mauritiana*	100% up to 60 days	Reduced growth	[41]
		*Holothuria fuscogilva*	100% up to 30 days	Necrosis	[22]
		*Holothuria scabra*	100% up to 10 days	Side effects not detected or not studied	[42]
		*Holothuria whitmaei*	100% up to 21 days	Necrosis, mark disappears, increased mobility in the field	[17]
Internal tags	Coded wires (in coelomic cavity and body wall)	*Actinopyga echinites*	100% after 63 days	Side effects not detected or not studied, difficult to use in the field, individuals need to be sacrificed	[43]
		*Holothuria fuscogilva*	60% after 63 days	Side effects not detected or not studied, difficult to use in the field, individuals need to be sacrificed	[43]
		*Holothuria scabra*	33–53% after 1 year	Mortality, difficult to use in the field, individuals need to be sacrificed	[24]
		*Parastichopus californicus*	37% after 224 days	Increased mobility in the field, difficult to use in the field, individuals need to be sacrificed	[16]
		*Thelenota ananas*	100% after 63 days	Side effects not detected or not studied, difficult to use in the field, individuals need to be sacrificed	[43]
	PIT tags in coelomic cavity	*Actinopyga miliaris*	0% after 8 days	Side effects not detected or not studied	[29]
		*Holothuria whitmaei*	25% after 8 days	Side effects not detected or not studied	[29]
	PIT tags in tentacles	*Cucumaria frondosa*	92% after 30 day and 68% after 300 days	See text	Present study
	PIT tags in body wall	*Cucumaria frondosa*	41% after 30 days and 33% after 300 days	See text	Present study
Chemical tags	Visible implant elastomers	*Cucumaria frondosa*	80% after 140 days	Side effects not detected or not studied, difficult to use in the field, no unique identifier	[32]
	Fluorochrome in ossicles	*Holothuria scabra*	100% after 1 year	Mortality, reduced growth, increased burying behaviour, toxicity, no unique identifier, unstable in sunlight and cold water	[23,24,29]
Genetic tags	DNA	*Holothuria whitmaei* (as *H*. *nobilis*)	No retention rate	Difficult to use in the field, expensive	[20,26,27]

Alternatively, chemical, genetic and internal tags have been developed. Chemical tags such as fluorochromes involve exposure to tetracycline or calcein which are incorporated in the carbonate structure of ossicles [23,24]. Although the technique is inexpensive, simple and lasting, it does not provide unique identifiers. Furthermore, these chemicals can be toxic, especially for juveniles, the amount of stained ossicles declines over time, and microscopy is required for reading, which makes it unsuitable for a number of applications [24,25]. Importantly, this technique may not be suitable for cold-temperate and polar species, because the uptake of fluorochromes is temperature-dependent [23]. Genetic markers are effective; however, they are impractical for short-term studies, generally expensive, time-consuming, unsuitable for field monitoring and they require extensive analytical skills [20,26,27]. As for internal tags such as coded wire tags (CWTS) injected in the body wall or in the coelomic cavity, they must be excised for identification, precluding repeated readings, because the individuals are usually sacrificed [16].

Passive integrated transponder (PIT) tags have been used successfully in several vertebrates and were recently expanded to marine invertebrates [28–30]. PIT tags are tiny inert microchips with an electromagnetic coil encapsulated in glass and a unique code identifier [30]. However, the presence of PIT tags in the coelomic cavity of green sea urchins, *Strongylocentrotus droebachiensis*, was associated with lower growth, gonad index and survival rates compared to controls [31]. To our knowledge, only one study has tested PIT tags in sea cucumbers; the tags were injected through the body wall into the coelomic cavity and resulted in low retention rates [29]. The possibility of placing PIT tags in other locations and the long-term effects of these tags on sea cucumber health and behaviour remain untested [16,17].

Tagging techniques tested on *C*. *frondosa* so far include various T-bar tags and dyes in the form of visible implant elastomers (VIE), with maximum retention rates of 65% and 80%, respectively, after 140 days [32]. Like fluorochromes, VIE have limited use for individual identification, which can only be achieved by varying the number of implants and their colour combinations. Moreover, long-term applicability was not examined and side effects were not investigated in depth; they are suspected to be consistent with previous studies of similar methods (Table 1).

The aim of the present study was to determine if and how PIT tags could be used as a reliable and innocuous marking technique in sea cucumbers. The efficacy of PIT tags implanted in various ways into previously unstudied tissues/organs was tested in two size classes of *C*. *frondosa* by evaluating retention rates, location of implanted PIT tags and post-tagging side effects on the body wall and on feeding and spawning behaviour during a short (30 d) and long-term experiment (300 d).

## Materials and Methods

### Sea cucumber collections

Large adult sea cucumbers with average (± sd; n = 120) immersed weight [9] of 11.7 ± 1.5 g, measuring 14.8 ± 1.3 cm contracted body length were collected by a fishing vessel (commercially licensed by Fisheries and Oceans Canada; DFO) on the southwest Grand Banks of Newfoundland (46°20’43.5” N: 56°23’0.28” W), eastern Canada, at depths between 20 and 30 m. Smaller individuals with average immersed weight of 2.6 ± 1.1 g and measuring 9.6 ± 2.8 cm (n = 60) contracted body length were collected by divers in Logy Bay (Avalon Peninsula, 47°37’39.6” N: 52°39’51.4” W), at depths between 5 and 10 m. Dive collections were performed by the Field Services of the Department of Ocean Sciences with the required DFO permits. Large and small sea cucumbers were kept in separate 500 L flow-through tanks in ambient seawater (temperature ~2°C and salinity of 35) for over a month before using them in any trial. Only healthy undamaged individuals exhibiting unblemished tegument, firm attachment to the substrate and regular tentacle deployment and retraction (i.e. normal feeding activity) were selected for tagging trials. Natural planktic food present in ambient seawater was available to sea cucumbers during the study.

### Tagging procedures and experimental conditions

For all experimental trials described below, sea cucumbers were tagged while half submerged in a tray filled with seawater at the same temperature as that measured in the experimental tanks. The latter consisted of 24 L containers supplied with ambient running seawater, at a flow of 10 L h^-1^. Over the course of the study, the water temperature ranged between 2.5 and 12.5°C, following seasonal fluctuations in the field. Light was provided through large windows and complemented by fluorescent lights to a maximum intensity of 200 lux following natural photoperiod which ranged from 8L/16D in winter to 15L/9D in summer. The various treatments and controls (detailed for each trial below) were randomly distributed in the experimental tank system.

Certified passive integrated transponder (PIT) tags measuring 8.21 ± 0.05 mm long, 1.29 ± 0.01 mm wide and weighing 29 ± 0.3 mg (AB10320) were purchased from Loligo Systems (Denmark) together with implanters (AB10490), a handheld reader and an external waterproof antenna. Implanters and PIT tags were sterilized with ethanol 100% before the procedure and all tags were tested for readability, both before and immediately after being implanted in the sea cucumbers.

### Experimental designs and data analysis

#### Preliminary experiment

A preliminary experiment was conducted in order to test three basic implanting locations as well as tag readability immediately after the procedure. Five large individuals were tagged into the coelomic cavity as per the only previous study using PIT tags in sea cucumbers [29], 5 individuals were tagged dorsally directly in the body wall just underneath the tube feet row, and 5 individuals were tagged through the body wall directly into the aquapharyngeal bulb. Tags in the coelomic cavity were implanted dorsally at mid-body length and released as soon as the implanter had punctured the body wall and the muscle band. However, these tags were not easily read (as they could move freely inside the body cavity), which made them unsuitable for routine post-tagging identification, and they were generally expelled within 48 h. The coelomic cavity treatment was therefore not retained in subsequent trials. Based on this preliminary experiment, a short-term experiment was devised using variations of the two most promising tagging locations (body wall and aquapharyngeal bulb).

#### Short-term experiment (30 days)

The short-term experiment consisted of 5 treatments (n = 12 sea cucumbers each) monitored for 30 days. The whole tagging procedure (for all individuals and treatments) was completed inside 2 h (taking an average of 2 min per sea cucumber). Only large sea cucumbers (size range provided above) were used. They were distributed in 30 tanks, using 6 tanks per treatment and 2 individuals per tank, for a total of 60 individuals. Treatments consisted of: (1) tagging in body wall, (2) tagging in aquapharyngeal bulb, (3) control for tagging procedure in body wall, (4) control for tagging procedure in aquapharyngeal bulb and (5) handling control. In the first treatment, sea cucumbers were tagged dorsally in the body wall (TBW) underneath the row of tube feet between the epidermis and the longitudinal muscle band at mid body length. Care was taken not to release the tag in the coelomic cavity or to implant it too superficially in the tegument where it could tear the epidermis and be lost or rejected rapidly, although the tissue layer in which the tags were implanted was not confirmed until the sea cucumbers were later dissected. In the second treatment, sea cucumbers were tagged directly in the aquapharyngeal bulb (TAB), by inserting the implanter 1 cm posterior to the oral cavity, at an angle of ~45°. For this treatment, the tag was released immediately after the implanter had punctured the body wall and a second puncture was felt, suggesting that the aquapharyngeal bulb had been reached (however there was no immediate confirmation that the tag was in the aquapharyngeal bulb rather than in the digestive tract or coelomic cavity). Treatments 3 to 5 were devised to control for the effects of puncturing or handling the sea cucumbers. The third treatment consisted of sea cucumbers punctured in the body wall (PBW), as in treatment one, without any tag being released. The fourth treatment comprised sea cucumbers punctured in the aquapharyngeal bulb (PAB), as in treatment two, but not tagged. Finally, the fifth treatment (Control) consisted of an overall handling control as sea cucumbers were neither tagged nor punctured but simply handled as the implanted sea cucumbers (placed in the surgical tray for 2 min and transferred to the experimental tank).

Apart from tag retention, side effects such as contraction of the entire body and appearance of unusual tension such as ripples in specific areas of the body wall were noted, together with the duration of such effects. Evisceration and lesions, as well as other morphological, physiological and behavioural responses that can provide an indication of stress or suboptimal health in sea cucumbers were monitored. Healthy sea cucumbers were expected to anchor firmly to the substrate, to respond to food by deploying their tentacles, to display the typical escape response to their main predator, and to release gametes during the spawning season. The main indicators that were routinely recorded are described in Table 2.

**Table 2:** Morphological, physiological and behavioural indicators of sea cucumber health monitored during the present study.

Indicator	Description	Tested in
Ripple	Small undulation visible on the sea cucumber body at the site of implantation or puncture (Fig 4A).	Short and long-term experiments
Skin lesion	Occurrence of tissue damage usually visible as different coloration than the surrounding tissue; may be the result of infection or immune reaction.	Short and long-term experiments
Anchorage	Firm attachment to bottom or side of the tank with the ventral podia as determined when individual cannot be dislodged with gentle poking.	Short-term experiment
Swelling	Abnormal enlargement of the sea cucumber body into a balloon shape.	Short and long-term experiments
Contraction	Contraction of the entire body through the action of longitudinal muscle bands.	Short and long-term experiments
Elongation	Lengthening of the body caused by extension of the muscle bands.	Short and long-term experiments
Evisceration	Total or partial extrusion of internal organs such as intestine, gonads and/or respiratory tree.	Short and long-term experiments
Cloacal opening	Number of times cloaca opens and closes in given interval of time, as water circulates through the respiratory tree.	Cloacal opening and feeding behaviour experiments
Tentacle deployment (feeding)	When all ten oral tentacles are fully extended in order to capture food in the water and one tentacle is introduced into the mouth (Fig 4B).	Cloacal opening and feeding behaviour experiments
Spawning event	Presence of oocytes and/or spermatozoa in the tanks (Fig 4C and 4D).	Long-term experiment
Escape response	Initiation of reactions such as contraction, elongation and swelling in the presence of a predator.	Short-term experiment

Sea cucumbers were monitored every hour for the first eight hours post tagging, four times a day during the first week, three times a day in the third week and twice a day in the fourth week. On each occasion, still-implanted and shed tags were read, and side effects (if any) noted (Table 2). At the end of the 30 days, a natural predator of *C*. *frondosa*, the sea star *Solaster endeca* [9], was placed over each sea cucumber in order to assess and time its escape response [9,33], such as contraction, elongation and swelling, for 10 min (Table 2). Finally, at the end of the trial, a microscopic investigation was conducted on those individuals that had retained their tags. Tags in the body wall were located with the reader and the surrounding tissue was isolated. Dissections were conducted by slicing thin layers of the tissue until the tags were revealed. Tags in the aquapharyngeal bulb were localized by removing and dissecting the aquapharyngeal bulb. All dissections were conducted under a stereomicroscope (Nikon SMZ1500) coupled to a digital camera (Nikon DXM1200F). Pictures were taken and the exact position of the tag in the tissue layers was determined.

Logrank survival analysis (α = 0.05) was used to evaluate differences in PIT tag retention rates among treatments at intervals of 15 days [34]. The proportion of tags retained was estimated with the Kalpan-Meier estimator followed by multiple comparisons using the Holm-Sidak test [16]. In order to test the hypothesis that any post-tagging swelling was caused by the implanted tag itself instead of the tagging procedure, the total number of observations in which sea cucumbers displayed post-tagging swelling was compared among treatments after 30 d. Data on swelling response violated the assumptions for use of parametric statistics even after transformation. For this reason, Kruskal-Wallis one-way ANOVA on ranks (α = 0.05) was used to compare differences in these responses among treatments, followed by Tukey tests as appropriate. To test whether or not the implanted tag or the tagging procedure influenced the time needed by sea cucumbers to display an escape response toward the presence of a predator, one-way ANOVA was used to compare treatments. Reaction such as elongation was Log_10_ transformed to achieve normality (determined by Shapiro-Wilk test). Contraction of the body was not statistically analyzed, because all sea cucumbers immediately contracted their body when the predator was placed over them. In addition, swelling of the body, as a response to the predator, was also not analyzed, because it occurred in all treatments after the observation period of 10 min, when the sea stars were removed from the tanks.

#### Long-term experiment (300 days)

Based on the findings of the preliminary and short-term experiments, a long-term experiment (300 days) was conducted using and refining the most promising techniques and locations. Two treatments were devised, each with 30 large and 30 small sea cucumbers (size ranges previously outlined). The first treatment involved tagging 30 large and 30 small sea cucumbers dorsally in the body wall (LBW and SBW, respectively). The other treatment consisted of tagging 30 large and 30 small sea cucumbers in the aquapharyngeal bulb but via a deployed oral tentacle (LT and ST, respectively). The latter technique was developed to refine the aquapharyngeal bulb tagging method previously tested in the short-term experiment. Limited retention rates had been obtained due to improper tag placement; a new implantation method was developed to ensure that the tag found its way into the aquapharyngeal bulb via the oral tentacles. The deployment of feeding tentacles was elicited (within ~12 min) by adding live phytoplankton (*Chaetoceros calcitrans*) at a concentration of 5 x 10^5^ cell ml^-1^ to holding tanks. One tentacle was gently held with a flat edge tweezer and the PIT tag implanted at its base, helping the tag to find its way towards the aquapharyngeal bulb via the hydrovascular system (minimizing the possibility of implantation into the coelomic cavity or digestive tract).

Individuals were monitored for tag retention and side effects once a month for a total of ten months (300 days) using the same criteria as in the short-term experiment (Table 2). However, the occurrence of mortalities, skin lesions and evisceration was monitored daily. Since the long-term experiment encompassed the spawning season of *C*. *frondosa*, gamete release or the presence of either oocytes or sperm in the tanks was also noted.

X-ray photographs were taken of a subset of sea cucumbers that were still tagged after 240 days. Three individuals tagged in the body wall and three individuals tagged in the tentacles were photographed with a Lixi X-ray (Model PS-500 OS) to visualize the location of the PIT tags. The sea cucumbers were placed in trays filled with seawater from their holding tanks and positioned inside the x-ray machine. The photographs took about 2–3 min per sea cucumber. Using these images as guides, all other individuals that were still tagged at the end of the long-term trial (300 days) were dissected for a finer tag location analysis. Individuals tagged in the body wall were dissected as described in the previous section. Sea cucumbers tagged in the aquapharyngeal bulb via the tentacles were dissected by removing the aquapharyngeal bulb, taking care not to damage any structure. A visual evaluation of the aquapharyngeal bulb was carried out to detect any abnormality that might be associated with the implanted tags. Longitudinal cuts were then made until the tag was found.

PIT tag retention rates in the long-term experiment were compared among treatments at two intervals: day 30 (to link with the short-term experiment) and day 300 (at the end of the experiment) by using logrank survival analysis [34]. The proportion of tags retained was estimated with the Kalpan-Meier estimator followed by multiple comparisons using the Holm-Sidak test.

#### Monitoring cloacal openings and feeding behaviour

Selecting the methods that yielded the highest tag retention rates, this trial was designed specifically to measure the time needed for the sea cucumbers to recover normal rates of water change in the respiratory tree after the tagging procedure. Large sea cucumbers were either tagged or punctured in the body wall (TBW and PBW, respectively) and in the tentacles (TT and PT, respectively) using the techniques applied in the long-term experiment described above. They were compared with control sea cucumbers that were just handled without being tagged (Control). This experiment used 12 individuals per treatment (4 per tank). They were acclimated in the tanks 5 days prior to the onset of the experiment, and monitored for 3 days post tagging.

Cloacal movement was monitored as an indicator respiration rate, i.e. water circulation in the respiratory tree where oxygenation occurs [35], by counting the number of cloacal openings within 2 min every 5 h for 3 consecutive days. Monitoring started 10 h before tag implantation to determine baseline respiration rates in all individuals immediately before the trial.

The potential effects of PIT tags on the feeding behaviour was evaluated by adding phytoplankton to all treatments at the end of the 3-day trial and the number of sea cucumbers exhibiting tentacle deployment and insertion into the mouth (Table 2) was monitored 30 min and 1 h after stimulation. Live phytoplankton (*Chaetoceros calcitrans*) identified as a food source in *C*. *frondosa* [36] was used at the concentration of 5 x 10^5^ cell ml^-1^. The water flow was interrupted during this brief procedure to keep the concentration of algae high.

In order to test the hypothesis that the increase in respiration rates caused by the tagging procedure is short-lived, the number of cloacal opening per minute (respiration rate) of each sea cucumber was compared among treatments. Data violated the assumptions for use of parametric statistics even after transformation. For this reason, Kruskal-Wallis one-way ANOVA on ranks (α = 0.05) was used to compare differences in this response among treatments, followed by Tukey test, at intervals of 5 h. Also, to determine whether or not the implanted PIT tag affects the tentacle deployment of sea cucumbers, the total number of sea cucumbers deploying their tentacles as a positive feeding response was compared among treatments 30 min and 1 h after the addition of the phytoplankton in the tanks using one-way repeated measures ANOVA.

## Results

### Tag retention rates

**Fig 1:**
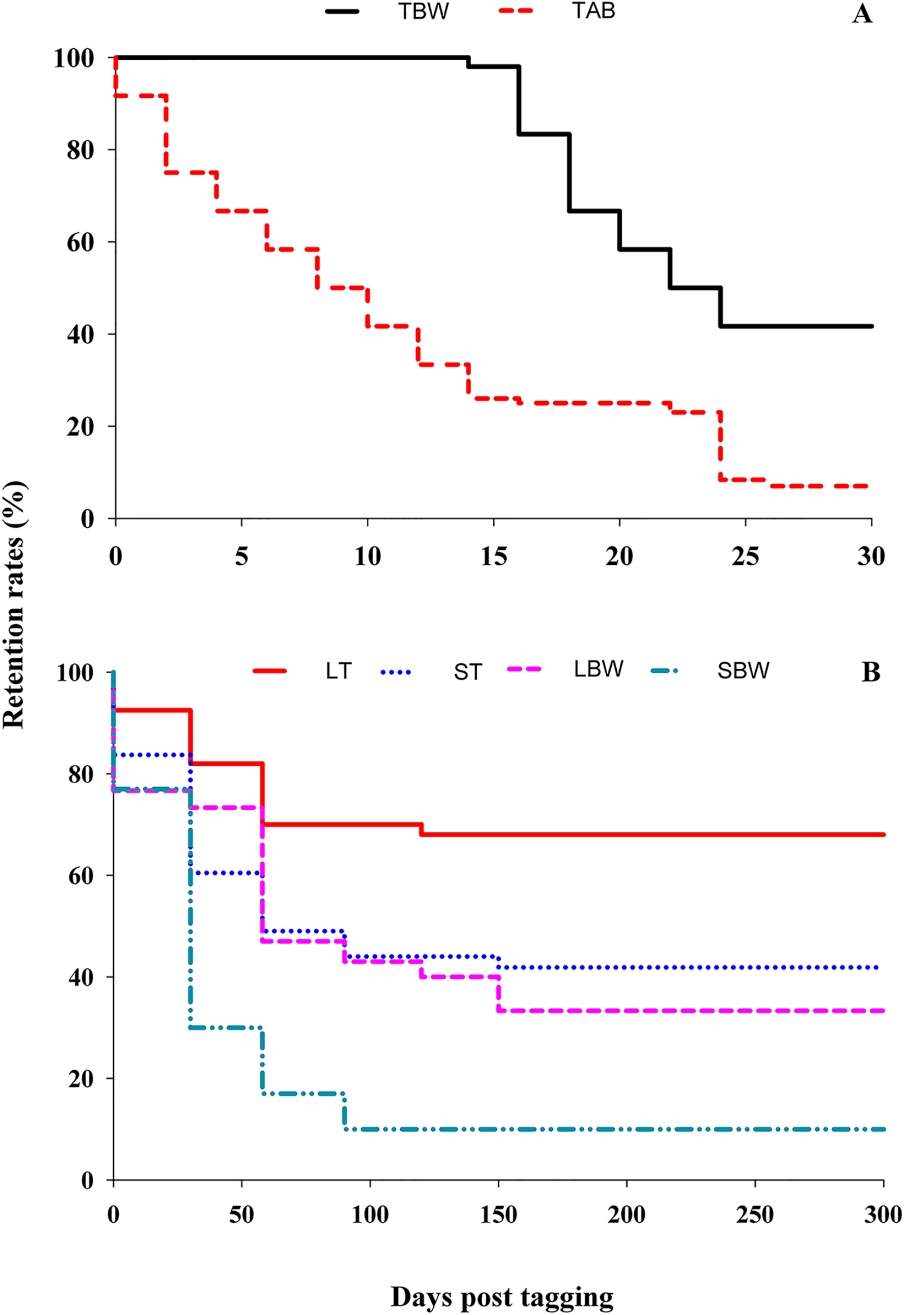
PIT tag retention rates in sea cucumbers. (A) Retention rates of tags implanted in the body wall and aquapharyngeal bulb during the short-term experiment. (B) Retention rates of tags implanted in the tentacles and body wall during the long-term experiment. TBW, tagged in body wall; TAB, tagged in aquapharyngeal bulb (directly); LT, large individuals tagged in tentacle (to aquapharyngeal bulb); ST, small individuals tagged in tentacle (to aquapharyngeal bulb); LBW, large individuals tagged in body wall; SBW, small individuals tagged in body wall.

In the short-term experiment, sea cucumbers tagged in the body wall had a retention rate of 100% in the first 15 days and 41% after 30 days (Fig 1A, S1 Table). Individuals tagged directly in the aquapharyngeal bulb started to shed tags in the day following implantation. Retention rate for this group dropped rapidly to 26% after 15 days (Fig 1A). There was no shedding over the following 9 days but only 8% of the sea cucumbers remained tagged for the full 30 days. Retention rates in the body wall were significantly higher than in the aquapharyngeal bulb after 15 days, although no difference between the two locations was noted 30 days post tagging (Table 3).

**Table 3:** Statistical comparison of PIT tag retention rates among treatments in the short-term and long-term experiments.

Experiment	Time	Retention rates among treatments	χ^2^	F	df	p
Short term	15 d	**TBW > TAB**	**8.000**		**1**	**0.005**
	30 d	TBW = TAB	1.648		1	0.199
Long term	30 d	**LT × ST × LBW × SBW**	**11.257**		**3**	**0.001**
		LT = ST		0.808		0.146
		**LT > LBW**		**9.468**		**<0.001**
		**LT > SBW**		**10.743**		**<0.001**
		ST = SBW		0.549		0.212
		LBW = SBW		0.208		0.648
		ST = LBW		0.513		0.276
	300 d	**LT × ST × LBW × SBW**	**233.137**		**3**	**<0.001**
		**LT > ST**		**53.364**		**<0.001**
		**LT > LBW**		**63.280**		**<0.001**
		**LT > SBW**		**225.767**		**<0.001**
		**ST > SBW**		**64.693**		**<0.001**
		**LBW > SBW**		**56.408**		**<0.001**
		ST = LBW		0.372		0.542

Results of Logrank survival analysis followed by Holm-Sidak test after 15 and 30 days (short-term) and after 30 and 300 days (long-term). TBW, tagged in body wall; TAB, tagged in aquapharyngeal bulb (directly); LT large individuals tagged in tentacle (to aquapharyngeal bulb), ST, small individuals tagged in tentacle (to aquapharyngeal bulb); LBW, large individuals tagged in body wall; SBW, small individuals tagged in body wall. Significant results are shown in bold.

Retention rates in the long-term experiment varied among treatments and were generally higher than during the short-term trial after the first 30 days (Fig 1B, S2 Table). Large sea cucumbers tagged via tentacles into the aquapharyngeal bulb exhibited the highest retention rate throughout the trial, i.e. 92% after 30 days and 68% at the end of the trial. For small sea cucumbers tagged in the tentacles the retention rate was 84% after 30 days and 42% at the end of the experiment (Fig 1B). In both large and small individuals, tag loss occurred only in the first 150 days whereas tag retention remained unchanged over the next 150 days. Retention rates for large sea cucumbers tagged in the body wall were 76% in the first 30 days and 33% at the end of the experiment. The retention rate of small sea cucumbers tagged in the body wall was the lowest measured in all treatments; it decreased quickly, reaching 77% at the end of the first month and dropping further to 10% after 300 days (Fig 1B). During the first 30 days, retention rates in large individuals tagged in the tentacles were significantly higher than in large and small individuals tagged in the body wall, but did not differ statistically from those in small individuals tagged in the tentacles. From day 31 until day 300, large sea cucumbers tagged in the tentacles had significantly higher retention rates than all other treatment groups (Table 3).

### Tag location

**Fig 2:**
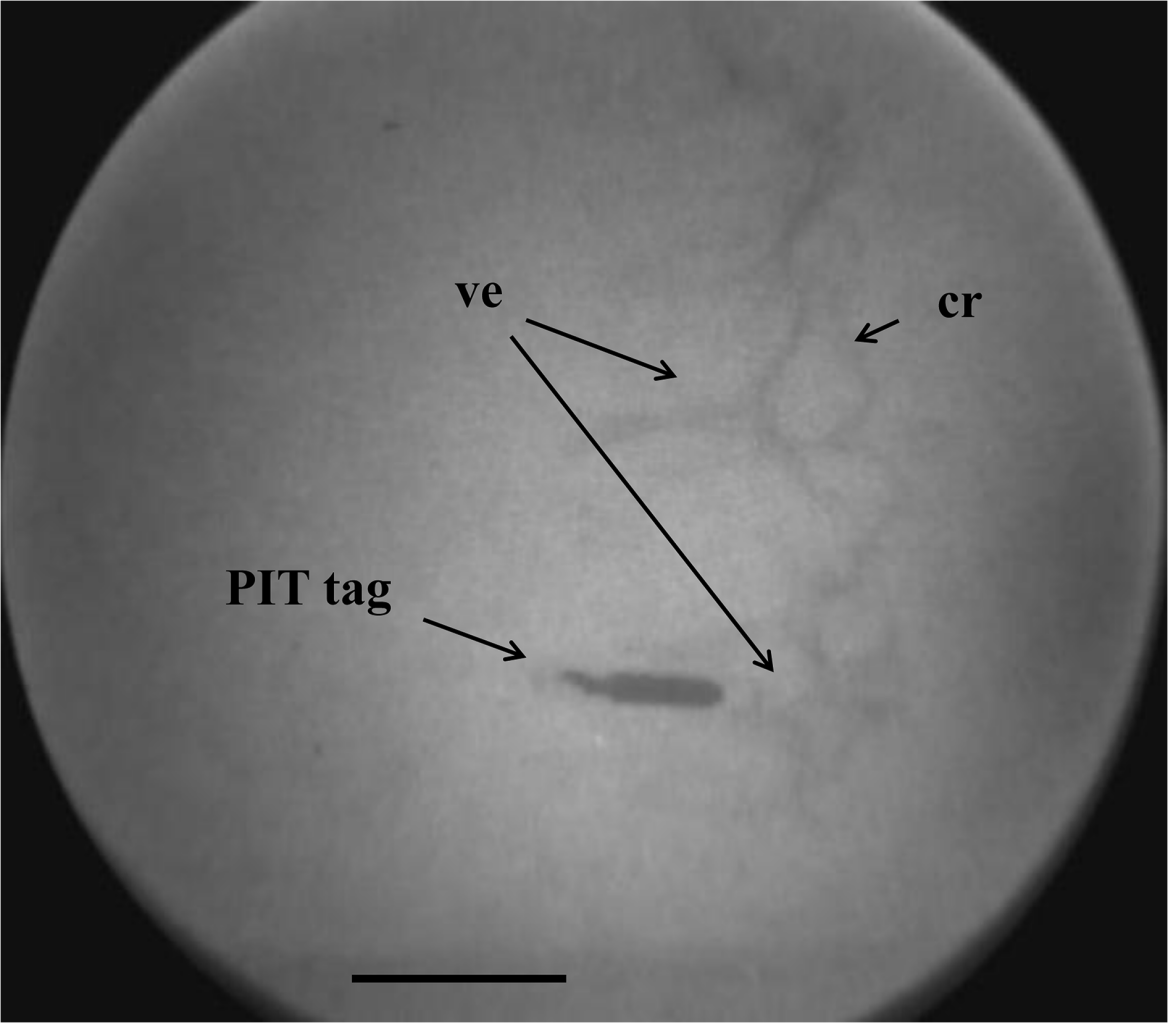
X-ray photograph of sea cucumber tagged in the aquapharyngeal bulb. PIT tags successfully implanted in the aquapharyngeal bulb through a deployed tentacle lodged themselves in one of the tentacle vesicles (ve) close to the calcareous ring (cr). Scale bars represent 2 cm.

X-ray photographs showed clearly that the tags implanted at the base of an oral tentacle were all lodged in the vesicle of the tentacle, inside the aquapharyngeal bulb (Fig 2). However, x-ray photographs of PIT tags implanted in the body wall were inconclusive given the absence of visually recognizable organs around them (all soft tissues).

**Fig 3:**
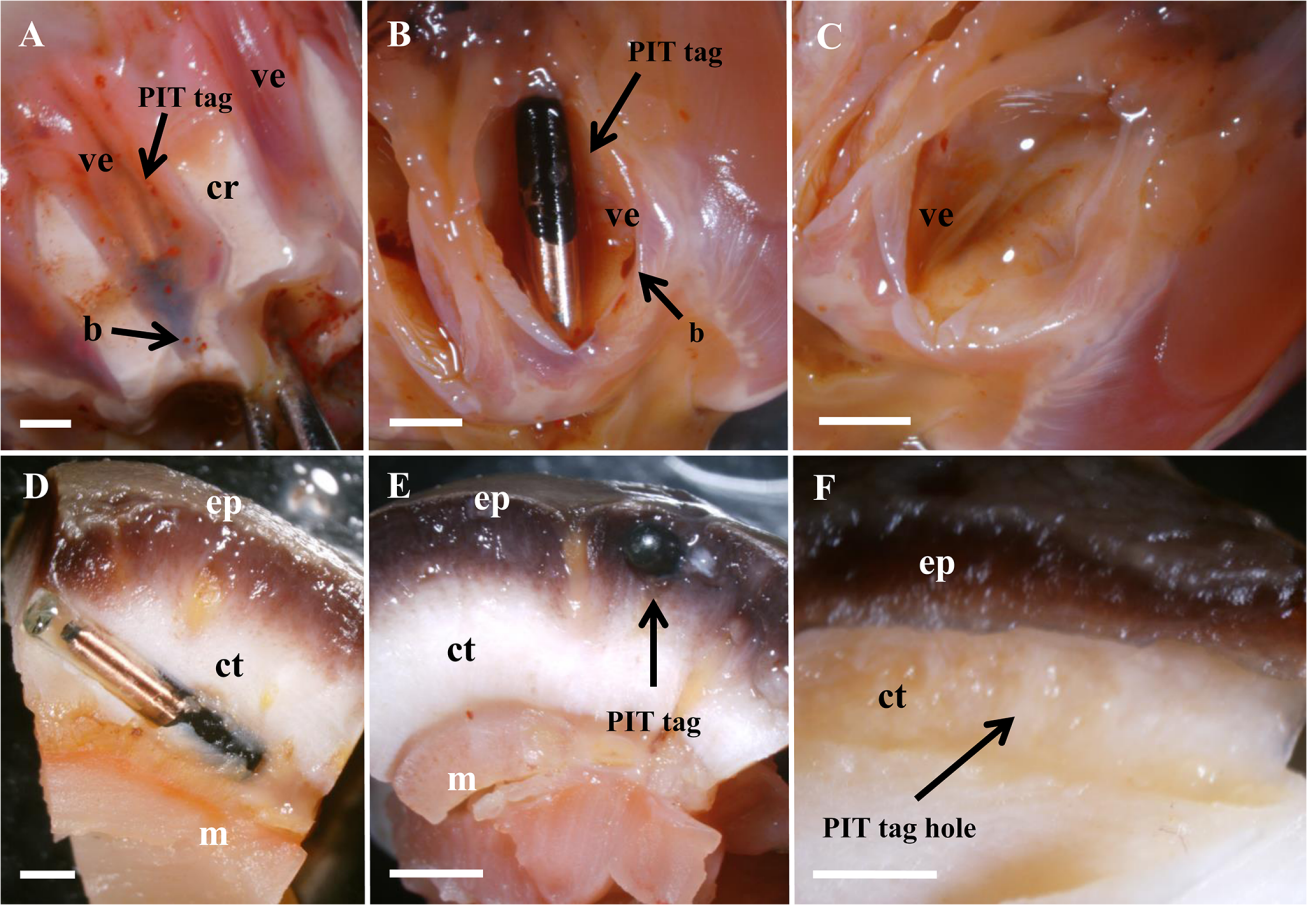
Localisation of retained PIT tags. (A-C) Tags retained in the aquapharyngeal bulb were found in the vesicles of the tentacles (ve). The calcareous ring (cr) and brown bodies (b) are identified. (D-F) Tags retained in the body wall were implanted in the connective tissue (ct) between the epidermis (ep) and the longitudinal muscle bands (m) below the ambulacral podia. Scale bars represent 2 mm.

Dissections confirmed that the tags implanted in the aquapharyngeal bulb via a tentacle were always located in the tentacle vesicle (Fig 3A). The tags were free in the vesicle, unattached to any tissue (Fig 3B). Tags that were retained in the body wall were implanted 1.3 ± 0.3 mm (n = 5) from the surface of the external epithelium, inside connective tissue (Fig 3D and 3E).

### Side effects of tagging

The tentacle vesicles where the PIT tags were found in the aquapharyngeal bulb were similar in terms of color and shape to the vesicles without tags. Brown bodies could also be seen in vesicles with and without tags (Fig 3A and 3B). No scars could be seen in the epidermis or in the connective tissue layers of individuals tagged in the body wall or in the aquapharyngeal bulb via the tentacles. No sign of either infection or abnormal cell development (e.g. proliferation of fibrous cells) was observed in the tissue surrounding the tags (Fig 3C and 3F).

**Fig 4:**
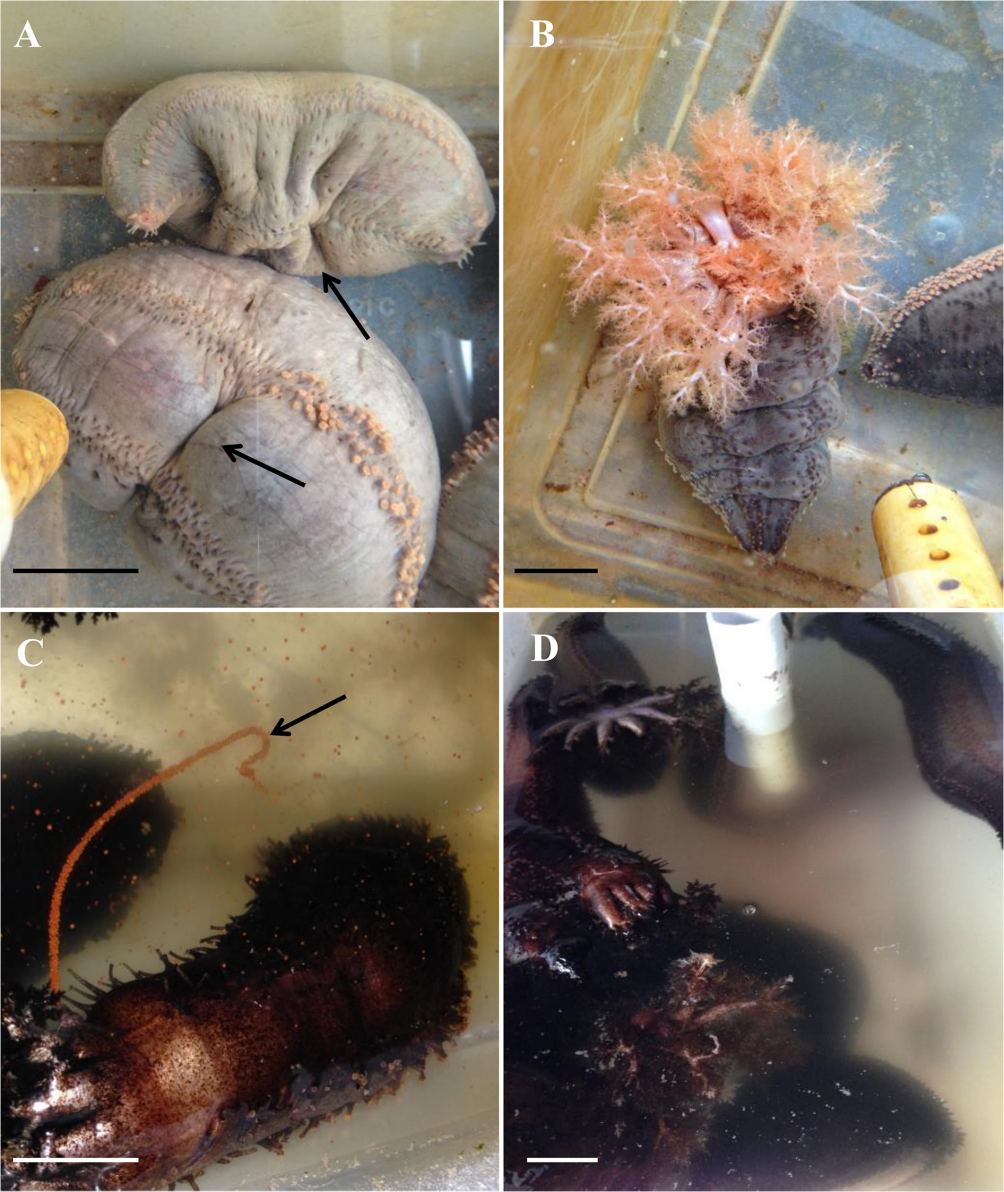
Minor side effects and normal behaviours recorded in tagged sea cucumbers. (A) Sea cucumbers tagged in the body wall showing ripples (arrows) around the implantation area immediately after tagging. (B) Large sea cucumbers tagged in the tentacles showed normal feeding, extending their tentacles fully and alternatively inserting them into the mouth. (C) A female sea cucumber tagged in the tentacles is releasing oocytes, visible as a reddish string (arrow), 40 days post tagging. (D) Water clouded with sperm in a tank holding sea cucumbers tagged in the body wall. Scale bars represent 3 cm.

Physiological and behavioural side effects were few; those that were noted appeared immediately after the tagging procedure and were short lived. The first observed side effect was a contraction of the entire body immediately after the puncture of the implanter, irrespective of whether or not a tag was inserted during the procedure. Individuals in the handling control group also showed the same contraction after being handled for measurements. Another nearly immediate side effect took the form of ripples along the body wall in 42 ± 9% of the sea cucumbers in the short-term experiment and 17 ± 6% during the respiration rate experiment (Fig 4A). This effect was limited to individuals tagged in the body wall. Sea cucumbers in all treatments managed to anchor themselves on the tank bottom and regain a normal posture 16.0 ± 1.2 min post procedure. About 30 min post tagging, swelling of the whole body occurred in 42 ± 3% of tagged and punctured individuals, but did not persist for more than 20 h and was only observed in the short-term experiment (S3 Table). In addition, there was no statistical difference among any of the treatments (H = 7.939, df = 4, p = 0.094).

**Fig 5:**
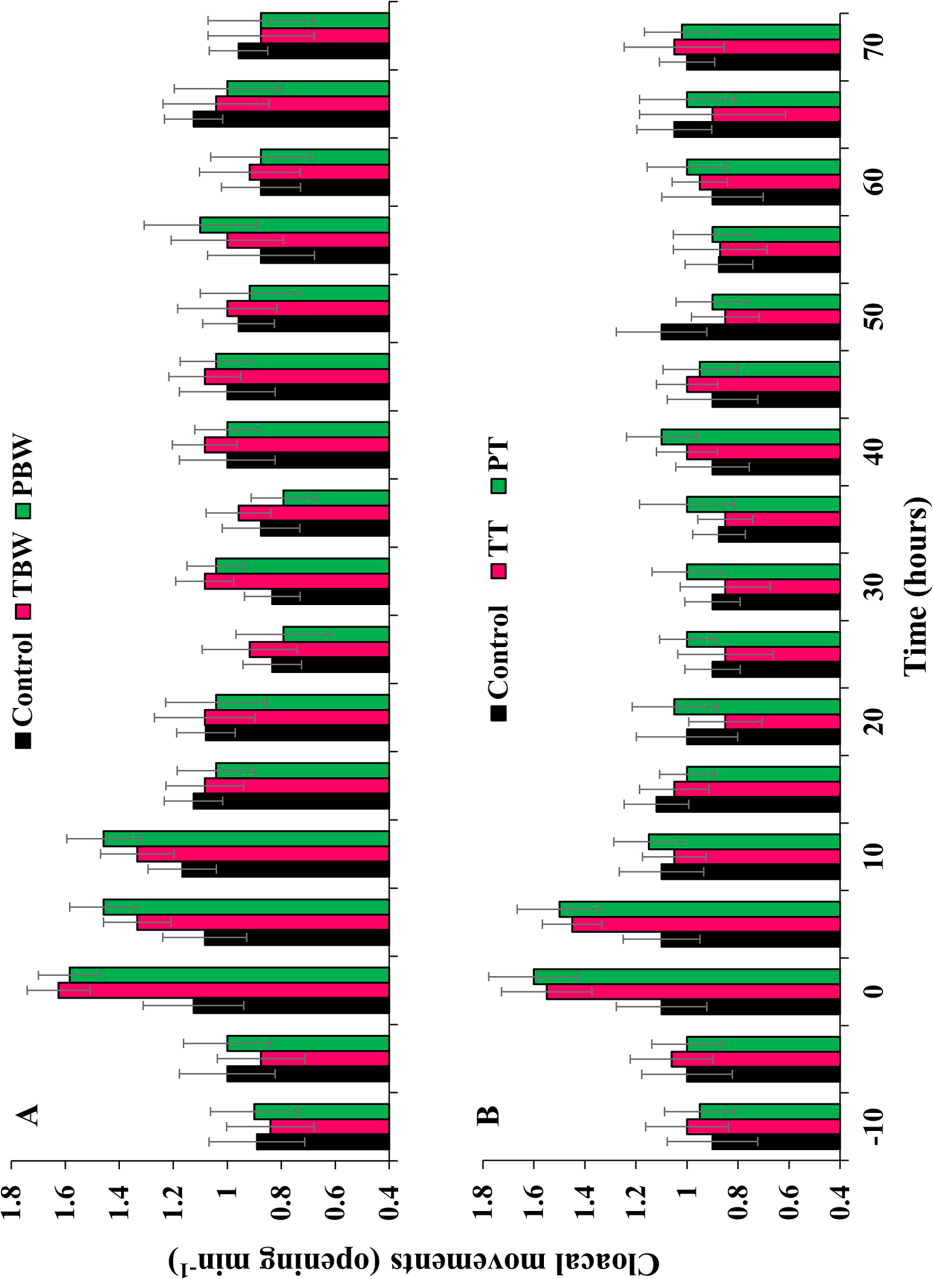
Cloacal movements of tagged, punctured and handled sea cucumbers. (A) Response of sea cucumbers tagged in the body wall. (B) Response of sea cucumbers tagged in the tentacles. Table 4 shows statistical results. TBW, tagged in body wall; PBW, punctured in body wall; TT, tagged in tentacle; PT, punctured in tentacle; Control, handled but not tagged or punctured.

Another common post-tagging behaviour was related to rates of cloacal movement, as a proxy of respiration rate (Fig 5, S4 Table). Acclimated sea cucumbers showed rates between 0.9–1.0 cloacal opening min^-1^ before the tagging procedure (time -10 h) and they did not differ statistically among treatments (Table 4). When sea cucumbers were either tagged or punctured, rates immediately increased to 1.6 ± 0.2 cloacal opening min^-1^ in individuals tagged in the body wall and to 1.5 ± 0.3 cloacal opening min^-1^ in individuals punctured in the body wall, whereas values in the control group remained lower at 1.1 ± 0.3 cloacal opening min^-1^ (Fig 5A). The increase was similar for tagged and punctured sea cucumbers in the body wall, both showing faster rates than the control group (Table 4). Similar results were found with individuals tagged and punctured in the tentacles, with post tagging increases to 1.5 ± 0.3 and 1.6 ± 0.2 cloacal opening min^-1^, respectively (Fig 5B; Table 4). Five hours post tagging, rates of cloacal movement exhibited by individuals tagged in the body wall were no longer different from the control group (Fig 5A). Cloacal movements of sea cucumbers punctured in the body wall stabilised to control levels within 15 h post tagging (Table 4). Values for individuals tagged and punctured in the tentacles levelled back to control levels 5 h earlier than sea cucumbers tagged or punctured in the body wall (Fig 5B). Fluctuations in cloacal opening rates were thereafter similar in all treatment groups (Table 4).

**Table 4:** Statistical comparison of cloacal movements (respiration rates) among treatments.

Experiment	Time (h)	Cloacal opening rates among treatments	H	df	p
Body wall	0	**TBW x PBW x C**	**17.382**	**2**	**<0.001**
		**TBW > C**			**0.006**
		**PBW > C**			**<0.001**
		TBW = PBW			0.857
	5	**TBW x PBW x C**	**22.565**	**2**	**<0.001**
		TBW = C			0.109
		**PBW > C**			**<0.001**
		TBW = PBW			0.104
	10	**TBW x PBW x C**	**26.42**	**2**	**<0.001**
		TBW = C			0.053
		**PBW > C**			**<0.001**
		TBW = PBW			0.077
Tentacles	0	**TT x PT x C**	**15.192**	**2**	**<0.001**
		**TT > C**			**<0.001**
		**PT > C**			**<0.001**
		TT = PT			0.887
	5	**TT x PT x C**	**10.107**	**2**	**0.006**
		**TT > C**			**<0.001**
		**PT > C**			**<0.001**
		TT = PT			0.834

Results of one-way repeated measures ANOVA on ranks, followed by Tukey tests. The increase in cloacal opening rates of tagged and punctured sea cucumbers in the body wall (TBW, PBW) and in the tentacles (TT, PBW) was compared to the control group (C) at various intervals post tagging. Sea cucumbers tagged at the base of oral tentacles recovered normal rates 5 h earlier than individuals tagged in the body wall. Significant results are shown in bold.

Addition of phytoplankton provoked an increase in tentacle deployment in sea cucumbers tagged in the body wall and tentacles, as well as in the control and punctured sea cucumbers (Fig 4B, S5 Table). The number of sea cucumbers with tentacles deployed did not vary significantly among treatments (F_4,49_ = 1.702, p = 0.242).

Measurements of reaction time in the presence of a natural predator at the end of the short-term experiment revealed that punctured and tagged sea cucumbers behaved similarly. When the sea star was placed on their dorsal surface, the first response was the contraction of the body, thereby increasing mid-body circumference and decreasing total length. The second response was the elongation of the body. The ANOVA analyses did not reveal any statistical difference among treatments in the time needed to initiate the escape response (F_4,55_ = 0.265, p = 0.899), which was on average 1.8 ± 0.3 min in all individuals and treatments (S6 Table). The final behaviour observed was the swelling of the body and production of mucus in all individuals from all treatments; this occurred as soon as the predator was removed. The effect lasted 30 to 40 min before sea cucumbers regained their original size in all treatments.

The long-term experiment covered the spawning season of *C*. *frondosa*. Sea cucumbers started to spawn 40 days after the tagging procedure. Fertilized oocytes could be observed in 60% of the tanks hosting sea cucumbers tagged in the tentacles (Fig 4C) and in 70% of the tanks with individuals tagged in the body wall (Fig 4D). Oocytes and sperm were seen in tanks hosting both small and large tagged sea cucumbers as well as in control tanks.

## Discussion

### PIT tag location, retention rate and readability

Tags implanted at the base of the tentacles (to reach the aquapharyngeal bulb) were most effective, with retention rates of 84–92% in the first 30 days, and 42–68% after 10 months. The calcareous ring and a series of valves that mediate the movements of the tentacles appear to trap the tag in the vesicle; individuals properly tagged in the aquapharyngeal bulb showed very stable tag retention, complete functionality of the tentacles during extension and retraction, and normal feeding. This stability presumably results from the fact that the well implanted PIT tags cannot escape from the vesicles. Conversely, tags lost during the first 150 days were likely not injected inside the tentacles and instead found their way into the coelomic cavity where preliminary experiments and other studies [29] showed poor retention rates (discussed below). Some tags may also have come out through the injecting hole [37,38]. The stable retention rates measured after 150 days suggest that this method should ensure tagging for several months or years, in addition to making the tags easy to read. This marking method is also innocuous (discussed below). Why retention rates were slightly lower in smaller individuals remains unconfirmed; it may simply be a matter of scaling. PIT tags used in the present experiment (~8 mm long) were the smallest available on the market at the start of the trials (advertised as 7 x 1.35 mm); however, they are likely too large to fit in the tentacle vesicles of some of the small sea cucumbers (~10 cm contracted body length), based on observations during dissections. This suggests that the eventual availability of smaller tags on the market should improve retention rates in smaller sea cucumbers. The efficacy of other physical (internal and external) tags has only explicitly been tested in full-size adults. Overall, PIT tagging at the base of the tentacles straight into the aquapharyngeal bulb emerges as a promising technique for conducting mark-recapture studies in *C*. *frondosa*, and possibly other sea cucumbers.

It should be noted that *C*. *frondosa* belongs to the order Dendrochirotida; it possesses 10 oral tentacles, which are fully extended in the water column during suspension feeding [36]. Most other commercial species of sea cucumber belong to the order Aspidochirotida and are deposit feeders with tentacles oriented towards the substrate (e.g. *Holothuria scabra*, *Isostichopus fuscus*, *Apostichopus japonicus*). While the latter generally have shorter oral tentacles, injecting tags at the base of the tentacles or in the hydrovascular system around the mouth should still be feasible and will be investigated in the near future. The size of tentacle vesicles will likely be the most important variable in determining the success (persistence) of PIT tags in various species and sizes of sea cucumbers.

Despite not being the most efficient in the long term, tags implanted in the connective tissue of the body wall exhibited retention rates of 100% over the first 15 days, making them very reliable for short-term studies. This technique is also among the easiest to use, and results in excellent tag readability. Several species of sea cucumbers possess thicker body walls than *C*. *frondosa*, which would make the procedure very simple. However, whether body wall thickness would necessarily improve tag retention remains uncertain. The tropical species *Holothuria scabra*, which possesses a very thick body wall, expelled T-bar tags within a month [24], although it should be noted that the later emerge externally, whereas PIT tags are fully buried in the tissues. Incidentally, placement of the PIT tags inside the body wall proved to be important. Microscopic examination of the persistent tags indicated that they were inserted superficially in the connective tissue and never in the longitudinal muscle bands, presumably preventing them from moving into the coelomic cavity, from which they can be expelled more readily.

Indeed, the least successful tag location in the present study was the coelomic cavity, where PIT tags were both difficult to read and expelled more rapidly. A previous investigation of PIT tag efficacy in two tropical sea cucumbers had only examined injection into the coelomic cavity, with similarly poor results [29].

### Side effects of tagging

Overall, the present study did not find any of the major disturbances reported with several of the marking techniques tested to date (summarized in Table 1). Dermal sores, skin sloughing, evisceration and death have been documented in sea cucumbers tagged with T-bars, scratches and brands [16,17,21]. The sores appear to be caused by T-bar tags that slip in and out of the body wall, causing stress and internal damage [16]. Fifty percent of *H*. *whitmaei* individuals tagged with T-bar developed infected wounds, whereas PIT tags implanted in the coelomic cavity did not elicit any detectable lesions [29]. On the other hand, a study with the green sea urchin *Strongylocentrotus droebachiensis* showed that PIT tags in the coelomic cavity resulted in lower rates of feeding, growth, movement, gonadal production and survival [28,31].

The rare side effects of PIT tags evidenced here in *C*. *frondosa* were short-lived (< 24 h), similar to minor effects reported when numbers were scratched on the dorsal body wall of *H*. *whitmaei* [17]. The contraction of the body observed in all treatments was akin to natural reaction following handling, and was therefore seen in the control group. Ripples around the tagging area were only observed in individuals tagged within the body wall, presumably the result of the recognition of foreign material, and disappeared within a few minutes. Tagging sea cucumbers in the aquapharyngeal bulb via the tentacles did not elicit this response, suggesting a less stressful implanting technique.

The most consistent side effect of PIT tag implantation in *C*. *frondosa* was the brief increase in rates of cloacal movement (as a proxy of respiration rate) until ~15 h post-tagging. Sea cucumbers tagged within the body wall and in the aquapharyngeal bulb through the tentacles showed very similar patterns. However, tagging sea cucumbers in the tentacles again seemed slightly less stressful, based on a more rapid return to baseline rates. Because punctured and tagged sea cucumbers showed similarly limited side effects, we assumed that the increase in cloacal movement (i.e. stress) was due to the puncture rather than the presence of the tag. Indications of increased metabolic activity after tagging have been evidenced: *H*. *whitmaei* with scratched numbers on the body wall, *T*. *ananas* with T-bar tags and *P*. *californicus* with 6 different tags showed increased mobility in the field during the first 78 h following marking, compared to sea cucumbers that were just handled [16–18]. Similarly, *H*. *scabra* marked with fluorescent dye increased their burying frequency in the first days after the procedure [23]. The need for more frequent renewal of water in the respiratory tree recorded here in *C*. *frondosa* and higher activity rates observed in other sea cucumbers suggest that stress can be induced by any physical disturbance akin to the attack of a predator, which was shown to increase respiration and movements of sea cucumbers in the field [17]. Increased activity was also reported in *C*. *frondosa* and other sea cucumbers exposed to predators in the laboratory [33,39]. However, in the case of PIT tagging, the reaction was shorter lived and dissipated quickly.

Another indication of the innocuity of PIT tags in the aquapharyngeal bulb and body wall of *C*. *frondosa* is the similar escape response displayed by tagged, punctured and control individuals in the presence of their natural predator, *Solaster endeca*. This is important information for mark-recapture and restocking studies, because tagged sea cucumbers should not be more vulnerable to a natural predator as a result of stress caused by the implanted microchip. Also, internal tags like PIT tags do not attract predator like T-bar tags may [17,21].

The presence of PIT tags in the aquapharyngeal bulb (more precisely in the tentacle vesicles) did not affect the feeding behaviour of *C*. *frondosa*; tagged and punctured individuals deployed their tentacles and moved them toward the mouth as consistently as the control groups. This is interpreted as a very positive sign since contraction/retraction of the tentacles is known to occur under stress [9]. Also, tagged individuals spawned during the same period as undisturbed sea cucumbers. Stress of capture, tagging and exposure to a new environment have previously been reported to inhibit the ability of sea cucumbers to spawn during the first few weeks in captivity [40]. However, the present study indicates that the presence of PIT tags does not have any major effect on feeding or gamete release, under the conditions and for the durations tested.

## Conclusion

PIT tags present a number of advantages over other marking techniques tested so far; chiefly, they are unique identifiers that can be repeatedly read with minimum disturbance (including underwater). While they are more expensive than some of the other tags, they are also reusable. The sea cucumber *Cucumaria frondosa* responded well, with either minor or no side effects, to the presence of PIT tags in most anatomical structures tested. While retention rates varied with the size of the individuals and the technique/location used to implant the tags, most previous studies have not explicitly investigated the effect of body size on tag efficacy, making it impossible to determine whether PIT tags present any advantage/disadvantage in this regard. Overall, implanting PIT tags at the base of the tentacles to reach the aquapharyngeal bulb emerges as one of the most effective techniques ever developed for tagging sea cucumbers reliably and innocuously for long periods, allowing individual marking and repeated identification without requiring emersion, elaborate analyses or lethal manipulations. Embedding PIT tags in the body wall also yields decent results, especially over short periods. In both cases, side effects were rare, minor and of short duration, and the presence of PIT tags did not affect feeding, spawning or escape responses to a natural predator. In addition, there were no records of developing wounds, necrosis or death as a result of tagging. Arguably, further investigations need to be carried out to confirm the suitability of the techniques outlined here in other holothuroids, including aspidochirotes. But taken together, the study provides promising data on the contextual efficacy of PIT tags and identifies means of minimizing side effects of tagging procedures in sea cucumbers. It will hopefully assist fishery, ecological and conservation studies and the sustainable development of sea cucumber aquaculture worldwide.

## Supporting Information

S1 Table Data file for tag retention rates in the short-term experiment (30 d). The data file is a comma delimited file and named “Table_S1.csv”. The first column, “Tag_N” gives the tag id; the numbers are preceded by “T” to insure entries will be read as character variables. The second column, “Treatments,” gives the location of the tag; TAB for tagged in the aquapharyngeal bulb, and TBW for tagged in the body wall. The remaining 70 columns give the time of observation; the first three characters identify the day (e.g., D01 for Day 1) and the final 5 characters identify the time of day (e.g., H0830 for 830 hours). Cell entries are “READ” or “SHED” for tag being retained and read or shed, respectively.
(CSV)

S2 Table Data file for tag retention rates in the long-term experiment (300 d). The data file is a comma delimited file and named “Table_S2.csv”. The first column, “Tag_N” gives the tag id; the numbers are preceded by “T” to insure entries will be read as character variables. The second column, “Treatments,” gives the size of sea cucumbers and the location of the tags; LBW and SBW for large and small sea cucumbers tagged in the body wall, and LT and ST for large and small sea cucumbers tagged in an oral tentacle, respectively. The remaining 10 columns give the observation period; the characters identify the month in which tags were read or recovered (e.g., M01 for Month 1). Cell entries are “READ” or “SHED” for tag being retained and read or shed, respectively.
(CSV)

S3 Table Data file for swollen body response. The data file is a comma delimited file and named “Table_S3.csv”. The first column, “Tank_N” gives the tank id; the numbers are preceded by “T” to insure entries will be read as character variables. The second column, “Treatments,” indicates whether sea cucumbers were tagged or punctured and the location; TBW for tagged in the body wall, PBW for punctured in the body wall, TAB for tagged in the aquapharyngeal bulb, PAB for punctured in the aquapharyngeal bulb, and Control for sea cucumbers just handled (neither tagged nor punctured). The third column, “Sea cucumbers”, indicates which sea cucumber showed swollen body behaviour after the tagging procedure. Cell entries are “Swollen” or “0” for sea cucumbers that displayed swollen body after tagging and sea cucumber that did not, respectively.
(CSV)

S4 Table Data file for cloacal opening response. The data file is a comma delimited file and named “Table_S4.csv”. The first column, “Tank_N” gives the tank id; the numbers are preceded by “T” to insure entries will be read as character variables. The second column, “Treatments,” indicates whether sea cucumbers were tagged or punctured and the location; TBW for tagged in the body wall, PBW for punctured in the body wall, TT for tagged in an oral tentacle, PT for punctured in an oral tentacle, and Control for sea cucumbers just handled (neither tagged nor punctured). The remaining 18 columns give the time of observation; the first character is identified as hour (H) and the final 3 characters identify the number of hours as before (e.g., -10 for 10 h before tagging) and after (e.g., +75 for 75 h after tagging) the tagging procedure. Cell entries are the number of cloacal openings per minute.
(CSV)

S5 Table Data file for tentacle deployment response. The data file is a comma delimited file and named “Table_S5.csv”. The first column, “Tank_N” gives the tank id; the numbers are preceded by “T” to insure entries will be read as character variables. The second column, “Treatments,” indicates whether sea cucumbers were tagged or punctured and the location; TBW for tagged in the body wall, PBW for punctured in the body wall, TT for tagged in an oral tentacle, PT for punctured in an oral tentacle, and Control for sea cucumbers just handled (neither tagged nor punctured). The remaining 3 columns give the time of observation. The first character is identified as hour (H) and the remaining 2 characters indicate hours after phytoplankton was added to the tanks. Cell entries are “Feeding” or “0” for sea cucumbers that displayed feeding activity and sea cucumbers that did not, respectively.
(CSV)

S6 Table Data file for response to presence of predator. The data file is a comma delimited file and named “Table_S6.csv”. The first column, “Tank_N” gives the tank id; the numbers are preceded by “T” to insure entries will be read as character variables. The second column, “Treatments,” indicates whether sea cucumbers were tagged or punctured and the location; TBW for tagged in the body wall, PBW for punctured in the body wall, TAB for tagged in the aquapharyngeal bulb, PAB for punctured in the aquapharyngeal bulb, and Control for sea cucumbers just handled (neither tagged nor punctured). The remaining 4 columns indicate the response to predator; C for contraction of the body wall, E for elongation of the body, D for doubling the original size, and S for swelling body. Cell entries are the time (s) needed for each sea cucumber to elicit each response.
(CSV)
